# A Single Glycine-Alanine Exchange Directs Ligand Specificity of the Elephant Progestin Receptor

**DOI:** 10.1371/journal.pone.0050350

**Published:** 2012-11-27

**Authors:** Michael Wierer, Anna K. Schrey, Ronald Kühne, Susanne E. Ulbrich, Heinrich H. D. Meyer

**Affiliations:** 1 Physiology Weihenstephan, Technical University Munich, Freising, Germany; 2 Leibniz-Institute for Molecular Pharmacology (FMP), Berlin, Germany; Univeristy of California Riverside, United States of America

## Abstract

The primary gestagen of elephants is 5α-dihydroprogesterone (DHP), which is unlike all other mammals studied until now. The level of DHP in elephants equals that of progesterone in other mammals, and elephants are able to bind DHP with similar affinity to progesterone indicating a unique ligand-binding specificity of the elephant progestin receptor (PR). Using site-directed mutagenesis in combination with *in vitro* binding studies we here report that this change in specificity is due to a single glycine to alanine exchange at position 722 (G722A) of PR, which specifically increases DHP affinity while not affecting binding of progesterone. By conducting molecular dynamics simulations comparing human and elephant PR ligand-binding domains (LBD), we observed that the alanine methyl group at position 722 is able to push the DHP A-ring into a position similar to progesterone. In the human PR, the DHP A-ring position is twisted towards helix 3 of PR thereby disturbing the hydrogen bond pattern around the C3-keto group, resulting in a lower binding affinity. Furthermore, we observed that the elephant PR ligand-binding pocket is more rigid than the human analogue, which probably explains the higher affinity towards both progesterone and DHP. Interestingly, the G722A substitution is not elephant-specific, rather it is also present in five independent lineages of mammalian evolution, suggesting a special role of the substitution for the development of distinct mammalian gestagen systems.

## Introduction

**Figure 1:**
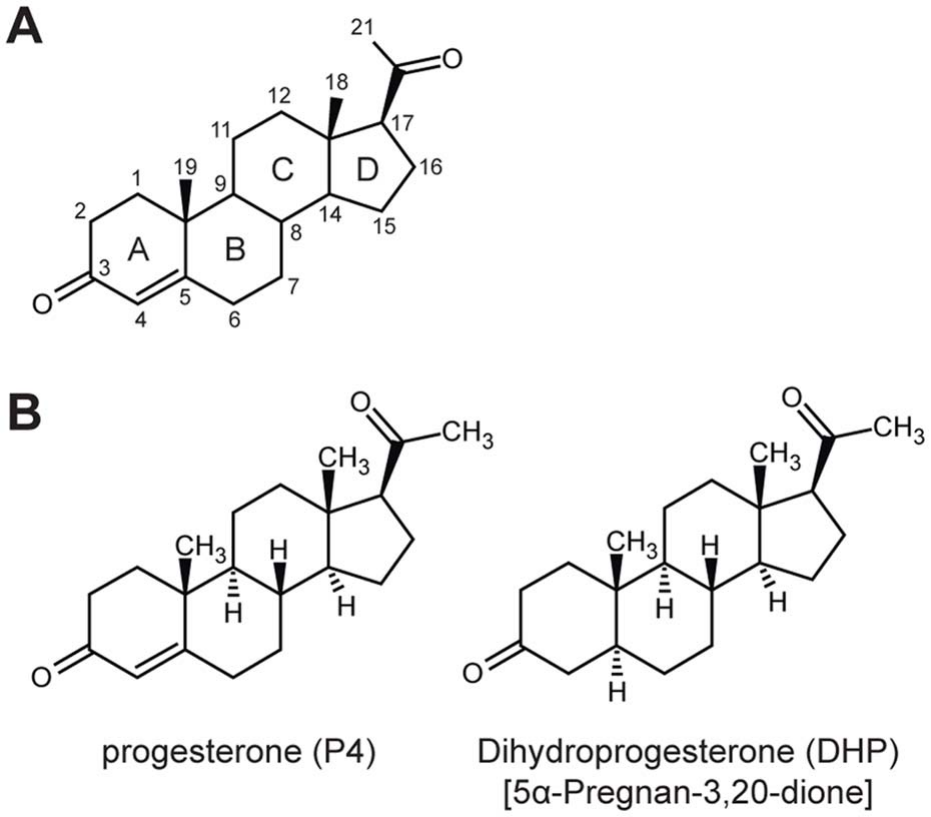
Progesterone and dihydroprogesterone differ in the reduction state of C4–C5. (A) Carbon atom and ring numbering system of steroids (B) Chemical structures of progesterone (P4) and 5α-dihydroprogesterone (DHP).

Gestagens acting via the progestin receptor (PR) serve as important mediators in the regulation of the ovarian cycle, and are responsible for maintaining pregnancy in mammals [1]. In most mammals studied so far the predominant gestagen is progesterone (P4), both in terms of blood levels and binding capacity of the PR [2]. By lacking progesterone at physiologically relevant concentrations, elephants are a unique exception. Progesterone blood levels of African (*Loxodonta africana*) and Asian (*Elephas maximus*) elephants are 100 to 1000-fold lower compared to other mammals and are therefore not able to serve as functional gestagen [3]. Furthermore, the concentration of progesterone neither changes during the ovarian cycle nor increases during pregnancy, indicating the lack of an endocrine role of progesterone in elephants [4], [5]. Searching for the relevant gestagen in elephants revealed high concentrations of the 5-alpha-reduced progestins 5α-dihydroprogesterone (DHP) and allopregnanolone, both being synthesized in the corpus luteum of the elephant ovary [5] (Figure 1). Serum levels of DHP show a close correlation with the ovarian cyclicity and remain constantly high from onset of pregnancy until parturition. While the binding capacity in mammals for DHP and allopregnanolone is generally low compared to progesterone, elephants can bind DHP with a similar affinity to progesterone indicating a change in the binding specificity of the PR [3].

The PR belongs to the group of steroid hormone receptors. These also include androgen receptor (AR), estrogen receptor (ER), mineralocorticoid receptor (MR) and glucocorticoid receptor (GR), which mediate crucial signals in reproduction, metabolism and salt homeostasis [6]. Steroid hormone receptors are part of the nuclear receptor family, which act as hormone-inducible transcription factors [6]. Upon hormone binding, activated PR is translocated to the nucleus, where it binds to specific cis-elements in the enhancer regions of target genes. DNA-bound PR recruits several cofactor complexes to specifically activate or repress the level of transcription [7]. Apart from its genomic actions, PR has also been shown to be involved in several signaling pathways including the MAPK and PI3K pathways through cross-talk with kinases located in the plasma membrane [8]. Despite the complexity of PR actions in the cell, the key event is the activation of PR by ligand binding, ruling over all subsequent molecular processes.

Steroid hormone receptors are modular proteins consisting of an N-terminal regulatory domain, a centrally-located DNA-binding domain (DBD) and a C-terminal ligand-binding domain (LBD) [9]. While the DBD is highly conserved between all steroid hormone receptors at the peptide level, the LBD shares conservation in structure and the amino acids flanking the binding pocket [10]. The specificity of LBDs from different steroid hormone receptors towards their respective ligands is based on hydrogen bond networks, hydrophobic interactions, as well as the steric size and shape of the binding pocket [9]. For instance, steroid hormone receptors which bind ligands with a 3-keto group (including PR, AR, MR and GR) have a conserved glutamine forming a hydrogen bond with the C3-keto group of the A-ring, while steroid receptors binding ligands with a 3-hydroxy group like the ER contain a glutamate at the corresponding position [10].

An example of how ligand specificity of steroid receptors evolved in detail was given for the GR, which evolved from a more promiscuous ancestral receptor that was activated by the mineralocorticoid aldosterone and more weakly by the glucocorticoid cortisol [11]–[13]. Two amino acid exchanges in helix 7 of GR specifically reduced the affinity for aldosterone and thus increased the receptor’s specificity for cortisol [11]. While the first exchange repositioned helix 7 closer to the ligand thereby reducing the affinity to all ligands, the second exchange generated a new hydrogen bond to the 17-hydroxyl group, which is unique to cortisol [12]. Additional substitutions in the GR LBD completed the loss of mineralocorticoid affinity, stabilized the new receptor conformation and generated an epistatic ratchet, which made the receptor’s evolution irreversible [13].

In this study, we address the unique ligand specificity of the elephant PR towards favored binding of DHP at the molecular level. Our approach consists of site-directed mutagenesis in combination with *in vitro* binding studies and molecular dynamics simulations. The latter method has been previously presented as a powerful technique to decipher the interaction between steroid hormone receptors and ligands without the restraints of a frozen picture provided by crystallographic analyzes [14], [15]. We furthermore elucidate whether the unique binding specificity and usage of DHP as a gestagen has a common background in mammalian evolution.

## Materials and Methods

### Ethics Statement

DNA samples from blood of Asian elephant (*Elephas maximus*), Przewalski’s horse (*Equus ferus przewalskii*), rhino (*Ceratotherium simum simum*), hyrax (*Procavia capensis*) and manatee (*Trichechus manatus)* as well as a vaginal mucosa tissue sample of the African elephant (*Loxodonta africana*) were obtained as a kind gift from the Institute of Zoo and Wildlife-Research in Berlin. Samples were taken post-mortem from recently deceased animals and have been approved by the Internal Committee for Ethics and Animal Welfare of the Leibniz-Institute for Zoo and Wildlife Research (IZW).

### Cloning and Bacterial Expression of Human and Elephant PR LBDs

Human PR LBD constructs were cloned from HOSE cell cDNA in a pET3d expression vector. For the initial experiments of stepwise introduction of elephant-specific amino acid exchanges in the human PR LBD the constructs involved amino acids 634–933 of human PR. For the following experiments a shorter fragment was cloned including aa 664–933 and an additional N-terminal MA extension. A corresponding region of elephant PR was cloned from vaginal mucosa cDNA. cDNA was generated from total RNA extracted with TRIzol (Invitrogen), which was reverse transcribed using M-MLV reverse transcriptase (Promega) according to the manufacturers’ instructions. Both inserts were sequenced and verified by comparison with the human PR transcript and elephant genomic sequence from the Genebank and Ensemble databases respectively. Site-specific mutations were generated using the QuikChange Multi-Site-directed mutagenesis kit (Stratagene).

PR LBD constructs were expressed in *E. coli* (BL21) cells in a 50 ml culture at 15°C overnight after induction with 0.1 mM Isopropyl-β-D-thiogalactopyranosid (Invitrogen). Cells were collected by 10 min centrifugation at 2000 g and resuspended in 3 ml assay buffer containing 40 mM Na_2_HPO_4_, 400 mM KCl, 0.5 mM EDTA, 1 mM DTT, 10% (w/w) BSA (Sigma) and protease inhibitor cocktail (Roche). Bacteria were lyzed using a French-Press and lysates were centrifuged at 24,000 g for 20 min (4°C) to remove cell debris and insoluble protein. The supernatants containing cytosolic proteins including the soluble PR LBD were frozen in liquid nitrogen and stored at −80°C until usage.

### Competitive Binding Assay

Bacterial lysates were diluted 1∶25 with assay buffer. 500 µl of diluted lysate was incubated with 1 nM [^3^H]-progesterone (Amersham Biosciences) and increasing concentrations of progesterone or DHP (both Sigma) for 16 h at 4°C. Unbound steroids were removed by adding 200 µl of 2% NoritA and 0.2% dextran (both Serva) in assay buffer to all tubes except the total-activity control, which received 200 µl assay buffer. Following 10 min incubation on ice, all tubes were centrifuged for 10 min at 3,000 rpm at 4°C. 100 µl duplicates of each supernatant were transferred into scintillation vials, mixed with 3 ml RiaLuma scintillation fluid (J.T. Baker) and measured in a scintillation counter.

Specific binding was calculated by subtracting non-specific binding ([^3^H]-progesterone completely displaced by adding 1 µM progesterone) from the amount of [^3^H]-progesterone bound to the receptor. SigmaPlot 8.0 (Systat software) was used to plot binding data, with IC50 values calculated using the one site competition setting.

### Molecular Modeling

All modeling procedures were performed on a dual-processor 3.06 GHz LINUX workstation or a LINUX cluster (48×3.2 GHz processors). The X-ray structure of human PR LBD with progesterone (PDB code 1a28) was used as a start structure [16]. Elephant PR LBD was constructed via homology modeling using the SYBYL 7.2 software and the B chain as a starting structure. Both receptor models (ligand extracted) were energy-minimized and used for docking of the energy-minimized ligands DHP and progesterone (automated docking and scoring with Surflex-Dock [17] SYBYL 7.2). The highest-scoring docking poses were minimized and used as input structures for subsequent molecular-dynamics (MD) simulations performed using SANDER (AMBER7). The simulations were performed under periodic-boundary conditions in a TIP3 water box (WATBOX216, minimal thickness 20 Å) with 2 fs time steps using weak-coupling temperature scaling and SHAKE. Ions were added for charge neutralization. The solvent was equilibrated under NTP conditions for 50 ps (isotropic pressure scaling) to reach constant box density. The production trajectories (constant volume) were run without any restraints for 5−20 ns. The development of the potential energy and of relative center-of-mass rms deviation of the Cα atoms from the start structure was monitored. Only the parts of the trajectories in which both values reached a steady state were subjected to further evaluation.

### Partial Sequencing of PR LBD from Different Mammals

Exon sequences comprising the PR LBD were amplified by PCR using degenerate primer pairs deduced from sequences of related species and sequenced. Exon-intron boundaries were amplified and sequenced following the Site Finding PCR protocol of Tan *et al.* [18]. The protocol was modified by adding a 1∶1000 dilution step after the first round PCR, and by directly using the purified product of the second PCR for the sequencing reaction. The genomic sequences were aligned with the sequence of human PR cDNA, using the ClustalW algorithm and the individual exon fragments combined to partial PR cDNA sequences (Figure S1).

### Phylogenetic Analyses

A phylogenetic tree of mammalian evolution was built using Dendroscope 3 [19] following phylogenetic studies of Murphy *et al.* [20] and Killian *et al.* [21]. PR-LBD sequences of human (NM_000926), mouse (NM_008829), rat (NM_022847), rabbit (M14547), cow (AJ557823), sheep (Z66555), pig (AJ245450, S49016), horse (AM158261), dog (NM_001003074), possum (DQ396888) and wallaby (S83227) were taken from the Genebank database. Sequence information from chimpanzee (CHIMP2.1), bushbaby (otoGar1), microbat (myoLuc1), megabat (pteVam1), tree shrew (TREESHREW), guinea pig (cavPor2), squirrel (speTri1), dolphin (turTru1), sheep (Ovis aries), panda (ailMel1), cat (CAT), armadillo (ARMA), tenrec (TENREC), African elephant (BROAD E1), opossum (monDom5), alpaca (vicPac1), sloth (choHof1), Tasmanian devil (DEVIL7.0) and platypus (Oana-5.0) were deduced from the genome sequences at the Ensembl database [22].

To test for positive selection of codons, we aligned the available PR LBD DNA sequences of all 35 mammalian species and performed an evolutionary codon analysis using the Selecton Server [23], [24]. Analysis was performed based on the phylogenetic tree of mammalian evolution and choosing the mechanistic empirical combination model (MEC) [25] with standard settings. Significance was determined by comparing the MEC test to the M8a null model. Positive selection was regarded to be significant, when the AIC score of MEC was lower than the AIC score of M8a.

## Results

### The G722A Substitution Changes the Ligand Specificity of the PR

**Figure 2:**
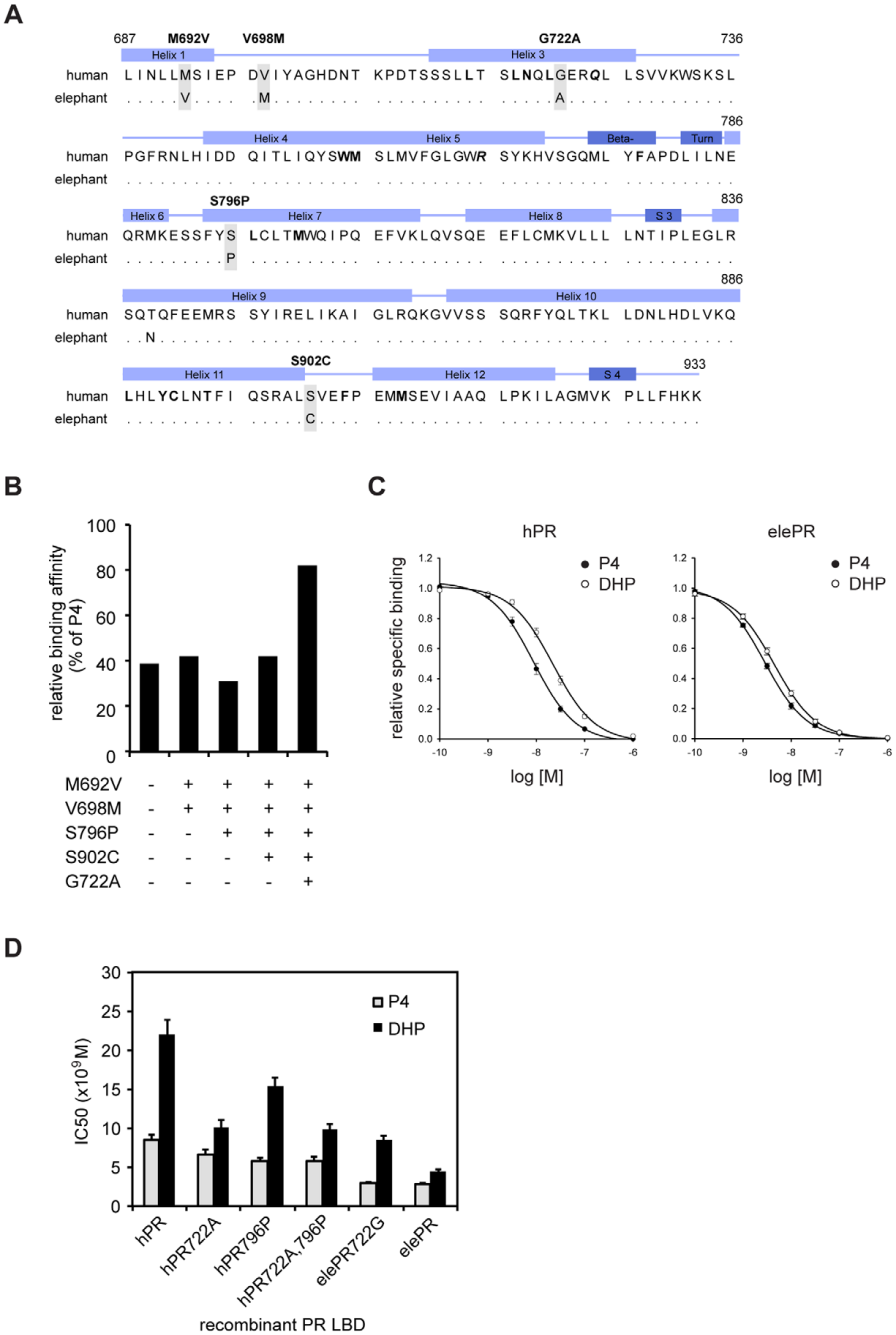
The G722A exchange alters receptor specificity of the PR. (A) The sequence of human PR LBD was aligned with the corresponding translated genomic DNA sequence of the African elephant (*Loxodonta africana*). Amino acids making van der Waals contacts with bound ligands are indicated in bold type, amino acids making hydrogen bonds to bound ligands are bold and italicized according to Williams *et al.* [16]. Secondary-structural elements of the PR LBD are indicated above the sequences. α-helices are pale blue, β-sheets and turns dark blue. Shaded residues indicate elephant specific amino acid exchanges. Dots resemble identical amino acids. (B) Elephant specific amino acid substitutions (+), were consecutively introduced into recombinant human PR LBD and relative binding affinity (RBA) of DHP compared to progesterone measured by competitive binding assays. (C) Competitive binding assays for progesterone and DHP with recombinant human (hPR) and elephant (elePR) PR LBDs. 1 nM [^3^H]-progesterone was displaced by increasing amounts of progesterone (P4) and DHP. (D) G722A and S796P exchanges were introduced into hPR, while A722G was introduced into elePR. IC50 values were measured as in (C). Data are presented as average IC50 values+SEM of at least three independent experiments.

In order to identify the molecular background of the altered binding specificity of the elephant PR, we aligned the amino acid sequences of human (hPR) and elephant (elePR) LBDs to find amino acid exchanges that potentially influence structure and ligand specificity of PR towards favored binding of DHP (Figure 2A). We identified 6 amino acid exchanges, none of which are involved in direct binding of the ligand according to the crystal structure of the PR-progesterone complex [16]. To examine, whether these amino acid changes are unique for the elephant PR and therefore might relate to favored binding of DHP, we aligned the elephant PR LBD with the corresponding sequences of pig, cow, dog, rabbit, rat and mouse (not shown); all mammalian species known to support pregnancy by the exclusive use of progesterone. Interestingly, the T839N exchange was present in all other species in the alignment as well, making it a human-specific exchange, while the other five substitutions appeared to be unique for the elephant PR.

To investigate the role of the five unique amino acid changes on binding affinity of progesterone and DHP, we set up an *in vitro* assay with bacterially expressed hPR LBD, in which the amino acid exchanges were consecutively introduced by site-directed mutagenesis. Stepwise introduction of M692V, V698M, S796P and S902C did not significantly change the relative binding affinity (RBA) of DHP compared to progesterone, indicating a lack of contribution to receptor specificity (Figure 2B). Strikingly, the introduction of the remaining G722A substitution in the four-fold mutated receptor increased the RBA of DHP 2-fold suggesting a key role in the change of elephant PR specificity (Figure 2B).

To verify whether the effect was solely due to the G722A exchange or a combination of several mutations, we introduced the mutation in the hPR alone and measured individual IC50 values for progesterone and DHP. While the G722A substitution only had a minor effect on the affinity of progesterone, the IC50 for DHP decreased 2-fold equaling a 2-fold increase in affinity (Figure 2D, left). We next cloned the elephant PR LBD from elephant vagina cDNA and performed the reverse mutation by exchanging Ala722 to Gly (Figure 2D, right). While the exchange did not affect progesterone affinity, the IC50 of DHP doubled. We therefore propose that the altered specificity of the elephant PR towards favored binding of DHP is solely due to the G722A exchange, whereas the other five exchanges do not have an effect on the receptor specificity.

Notably, by comparing the affinity of the G722A-mutated hPR with elePR for either progesterone or DHP, we observed a 2.3-fold higher affinity for elePR in both cases (Figure 2C, 2D). Therefore, apart from having an altered specificity, the elephant PR LBD also has an overall increased ligand affinity compared to hPR, which is likely mediated by additional amino acid differences between both species.

### Human and Elephant PR LBD Differ in DHP Positioning

**Figure 3:**
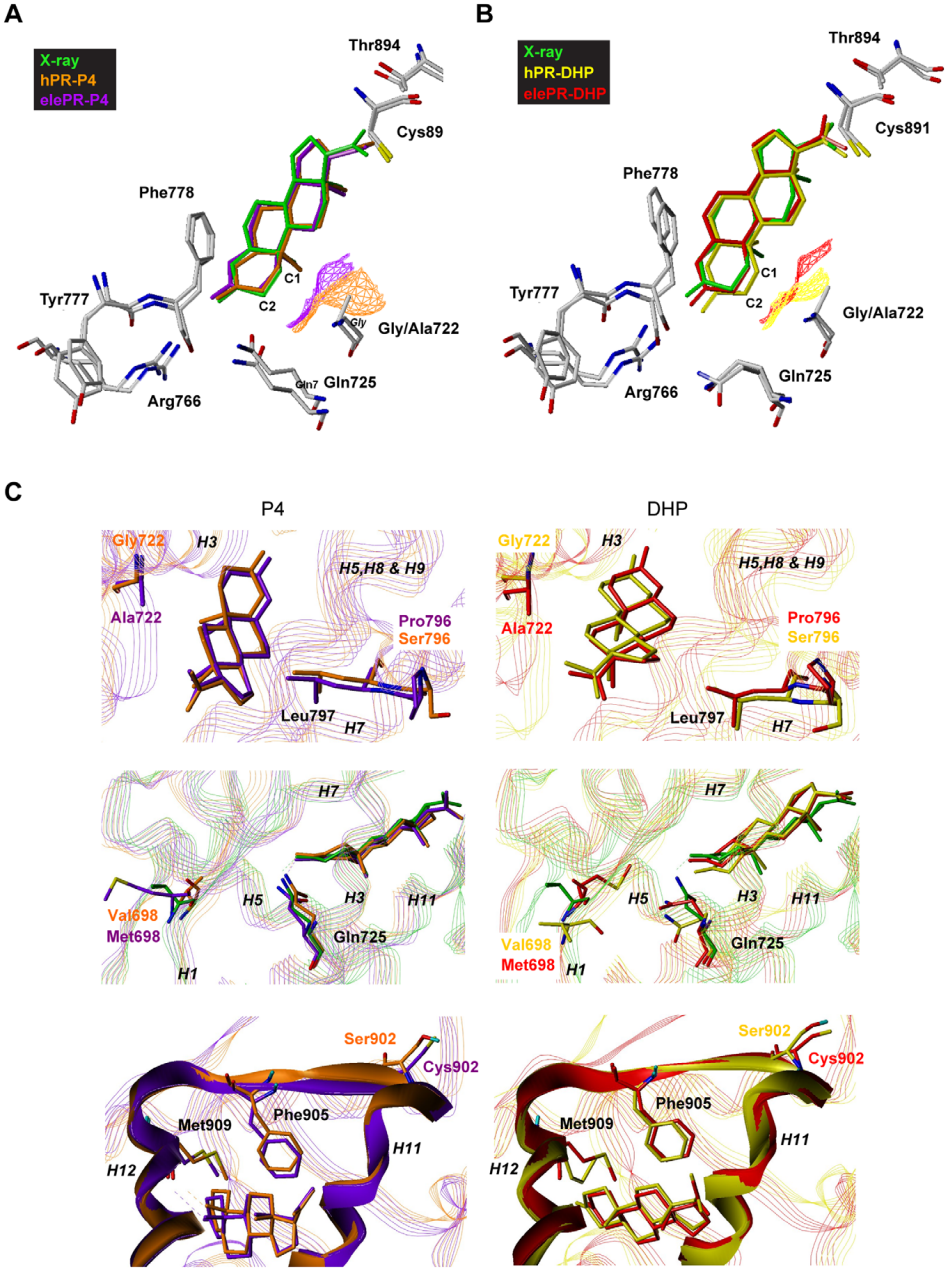
In elePR alanine 722 orientates the DHP A-ring into a position similar to progesterone. (A,B) Structures of progesterone (P4) and DHP bound to the human and elephant PR LBD compared to the X-ray structure (PDB code 1a28) (green) generated by molecular-dynamics simulations. The frames of each trajectory were fitted on the start frame using the Cα atoms of the helical parts and resulting averaged coordinates were used for a further fit. Represented are the frames with the least relative deviation to the averaged coordinates. The contour lines represent the solvent/ligand-accessible parts of residue 722. (A) hPR-P4 (orange), elePR-P4 (violet). (B) hPR-DHP (yellow), elePR-DHP (red). (C) Depiction of the four polymorphisms G722A, S796P, V698M, and S902C color-coded as in (A, B). Top panels: Depiction of G722A and S796P: Residue 722 and L797 form an axis through the binding pocket. Center panels: V698M is located in a flexible hinge and rotates freely towards and away from the binding pocket. Met but not Val can reach the crucial binding residue Q725. Bottom panels: Depiction of S902C, the hinge between H11 and H12, and the binding residues in the hinge and in H12.

To identify structural differences in the binding of progesterone and DHP to human and elephant PR, we performed unrestrained molecular dynamics simulations based on all four receptor-ligand combinations. This allowed us to study ligand positioning as well as local receptor flexibilities. The closest-to-average structures of the trajectories in comparison to the X-ray structure are shown in Figure 3. While progesterone docked in a position similar to the X-ray structure in both receptors (Figure 3A), DHP exhibited a slightly different orientation compared to the ligand in the crystal-structure (Figure 3B). The binding pocket is relatively restricted around the A-ring, leading to similar positions of the A-rings of both ligands. The ring geometries, however, differ between progesterone and DHP because of the double versus single bond between C4 and C5 (Figure 1). This causes a twist in the ligand leading to different positions of the remaining three rings of progesterone and DHP (seen best Figure 3C, center panels). Yet, the acyl group attached to the D-ring is found in a similar position in all four investigated complexes. Due to the altered geometry of the A-ring, there is no contact between C19 and the receptor. Therefore, DHP bound to hPR adopts a position with the A-ring that is closer to the H3 helix resulting in an A-ring orientation that is different from progesterone (Figure 3B). In the elePR-DHP complex this orientation is not possible, as the methyl side chain of Ala722 would clash with C1 of the ligand. As a result, the DHP A-ring in the complex with elePR is shifted away from the H3 helix, adopting an orientation that is similar to that of the progesterone A-ring.

### Different Positioning of the A-ring Affects Hydrogen Bond Network

The correct position of the A-ring in the binding pocket has a crucial impact on ligand binding. Tyr777, Phe778, Arg766, and Gln725 form a tight binding site for the A-ring of the ligand [14], [16]. Arg766 in H5 and Gln725 in H3 form a hydrogen-bonding network with O3 of the ligand and one water molecule. As reported earlier for PR and other steroid receptors, we found this water molecule to exchange with the bulk solvent [15], [26]. Additionally, Nε and Nη^2^ of Arg766 are hydrogen-bonded to the backbone carbonyls of Tyr777 and Phe778 in the β-turn. These hydrogen bonds form a clamp between the H5 helix and the β-turn of the receptor and are responsible for the positions of both the Arg766 guanidine group and the side chain of Phe778. The phenyl ring of the latter interacts with the A-ring of the ligand. Because of the two attachment points in the β-turn, the guanidine group can shift along an axis, allowing a certain tolerance in the optimal binding geometry.

**Figure 4:**
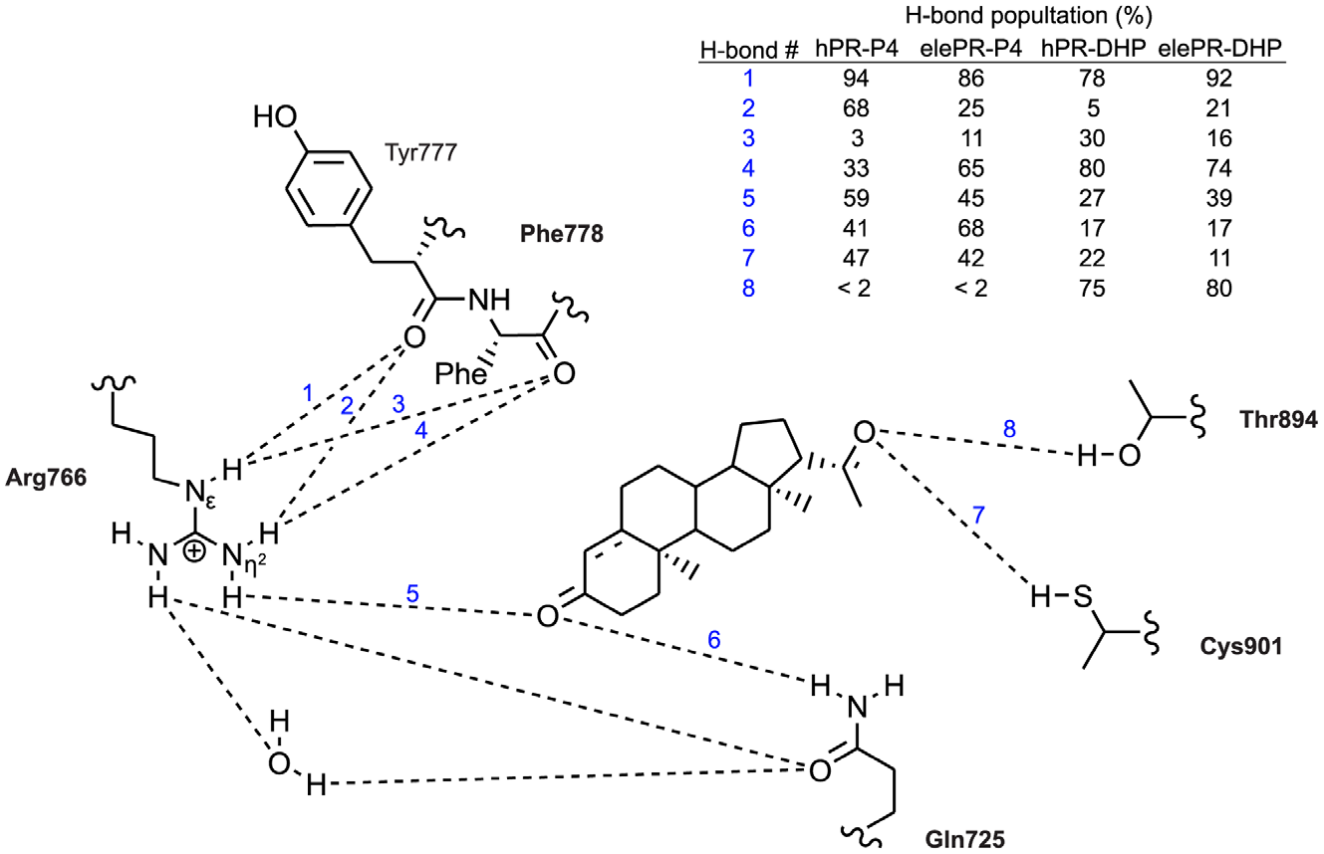
Different positioning of the A-ring affects hydrogen bond network. Hydrogen bond populations of the individual receptor ligand interactions were determined by molecular dynamics calculations for hPR-P4 (orange), hPR-DHP (yellow), elePR-DHP (red) and elePR-P4 (violet).

The altered position of the DHP A-ring in the hPR complex had two effects on the binding pocket. First, the orientation of the carbonyl group of the A-ring was less favorable for the hydrogen bond with Arg766 (Figure 4). While for the elePR-DHP complex we observed a H-bond probability between Arg766 and the O3 group of 39%, in the human receptor it only was present in 27% of all recorded frames. As a consequence of the lower H-bond population, the guanidine group of Arg766 in the hPR-DHP complex was shifted to a position very close to the backbone carbonyl group of Tyr777 and thus partially balanced the altered position of the A-ring carbonyl.

Secondly, the shifted orientation of the DHP A-ring in the hPR led to a destabilization of the hydrogen bond network between helix 5 and the β-turn. Considering the populations of the hydrogen bonds between Nη^2^ of Arg766 and the backbone carbonyls of Tyr777 and Phe778 respectively, for the hPR-P4 complex the resulting sum was 101% (Figure 4). However, in hPR-DHP, the summarized hydrogen-bond populations were only 85%. In the elePR complexes with both P4 and DHP these values were much more similar with 90% and 95%, respectively.

From our data we can conclude that the altered A-ring position in the hPR-DHP complex affected the hydrogen bond network of the binding pocket, both reducing the probability of H-binding via Arg766 as well as destabilizing the intra-residual H-bonds of the binding pocket.

### Additional Amino Acid Substitutions Increase Ligand Affinities of the PR Accompanied by a Loss of Flexibility of the Binding Pocket

We next sought to analyze the molecular basis of the overall gain of ligand affinity that we have observed for elePR compared to hPR, by focusing on the additional amino acid differences between both species. Three amino acid exchanges were found in close vicinity to the residues involved in steroid binding: V698M, S796P and S902C. The S796P exchange was particularly interesting to us, as it is located just before Leu797 known to bind the D-ring of progesterone. As shown in Figure 3C (top), both Ser and Pro in position 796 allow the adaptation of Leu797 to the altered D-ring position of DHP. To analyze whether Pro796 was involved in the gain of affinity to both progesterone and DHP, we introduced the substitution in the hPR and performed *in vitro* binding assays with the recombinant hPR796P protein (Figure 2D). We measured a 1.5-fold increase in affinity for both progesterone and DHP, indicating that the S796P exchange could at least partly account for the higher affinity of the elePR (Figure 2D). Interestingly, additional introduction of the G722A substitution in the hPR-S796P specifically increased DHP affinity without affecting the affinity of progesterone (Figure 2D). This further indicates the importance of Ala722 for the specificity of the receptor.

**Figure 5:**
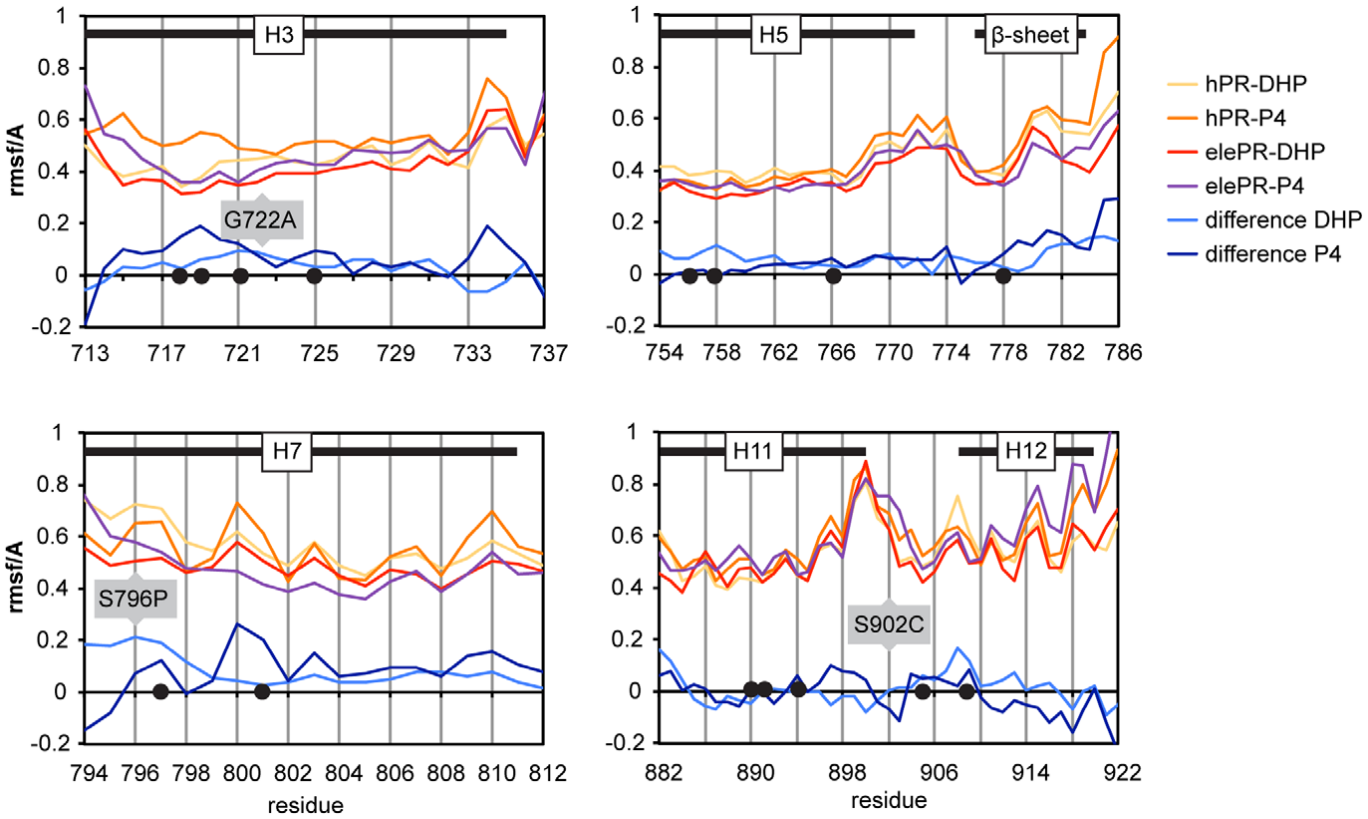
Gain of ligand-affinity in the elephant PR is accompanied by a loss of flexibility in the binding pocket. Flexibilities of the Cα atoms for the regions of the receptors that are involved in ligand binding, calculated as mean root mean square deviations from the averaged structure (rmsf/A) and difference curves between the hPR and elePR complexes of either given ligand DHP or P4. Residues involved in ligand binding are indicated with black dots.

Prolines have one fixed dihedral angle and therefore result in local rigidity of polypeptide chains. To study whether the S796P substitution would lead to a local gain of rigidity we analyzed flexibility of the binding pocket of the elephant compared to the human PR (Figure 5). Interestingly, for Leu797 we identified a slightly higher rigidity in elePR trajectories with both progesterone and DHP expressed as positive values of route mean square fluctuations (rmsf) of the Cα atom in the difference curves (Figure 5). We also observed a gain of rigidity for most other amino acids of the binding pocket, especially the helices 3 and 5 possibly mediated by the other substitutions (Figure 5).

The V698M exchange is located in the hinge between helix 1 and helix 3 and comes close to Gln725 in Helix 3, which binds to the C3 carbonyl. In the X-ray structure, Val698 is rotated away from Gln725 and therefore also from the binding pocket (Figure 3C, center). In the simulation, the hinge was flexible. Consequently, we also found orientations where the Val698 side chain projected in the direction of Gln725. In this case, the side chain of methionine but not of valine at position 698 was long enough to reach Gln725 and prevent the latter from rotation.

S902C is located on the C terminus of helix 11 and precedes Phe905, which interacts with the C20-keto group of the ligand. In the X-ray structure, its side chain protrudes into the bulk solvent, while in the simulation we also found H-bonds between Ser902 and the backbone (Figure 3C, bottom). As a result, we found a higher flexibility for the elephant receptor around this position (Figure 5). However, as the differences are rather small and differ strongly between ligands, the impact of this mutation on the hinge between helix 11 and helix 12 bearing the binding residues Phe905 and Met909 is difficult to predict.

### Elephant PR has Reduced Affinity for a Synthetic Gestagen

**Figure 6:**
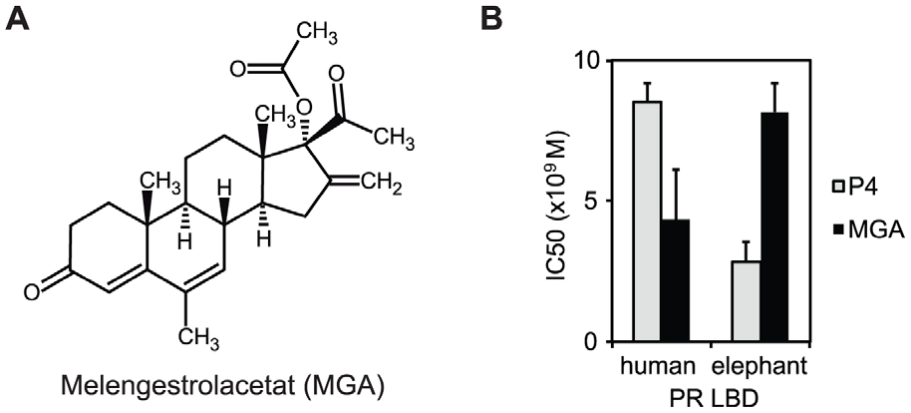
Increased binding affinity of the elephant PR cannot be generalized to synthetic progestins. (A) Chemical structures of melengestrol acetate (MGA). (B) IC50 values of MGA and progesterone (P4) binding to human and elephant PR were determined by competitive binding assays.

Having found the molecular mechanism by which elephant receptor changes its specificity for endogenous gestagens, we next tested the binding affinity of the synthetic gestagen melengestrol acetate (MGA). The binding affinity of MGA to the human receptor was 2-fold higher compared to progesterone (Figure 6B). Interestingly, the elePR responds differently, having a 2.9-fold smaller binding affinity of MGA relative to progesterone (Figure 6B).

Both G722A and S796P substitutions might play a central role in the binding affinities of MGA. MGA bears an additional unsaturation in the B ring, making the structure more flat than progesterone (Figure 6A). Furthermore, MGA has three additional side-chains, which anchor the structure in the binding pocket. In elePR, Ala722 narrows the binding pocket around the A-ring and the B-ring due to the methyl group. This might lead to sterical clashes with the C6 methyl-group of MGA. Additionally, a smaller negative effect could stem from the substituent at C16 being in contact with Leu797. Leu797 in turn is a residue whose environment is restrained by the S796P exchange.

Taken together, the increased binding affinity of the elephant PR to progesterone and DHP can therefore not be generalized to all gestagens, but is highly ligand-dependent.

### The G722A Exchange Evolved Under Positive Selection Five Times during Mammalian Evolution

Apart from elephants, which exclusively use DHP as gestagen during the ovarian cycle and pregnancy, also horses are known to regulate late pregnancy by high levels of DHP while progesterone levels are extremely low [27], [28]. Binding studies with horse (*Equus caballus*) uterine PR revealed high relative binding affinity for DHP similar to what has been observed for the elephant PR proposing a coevolution of horse and elephant PR [2]. Comparing the LBD of horse and elephant PR to the human sequence revealed only one common amino acid substitution, which was the G722A exchange (Figure S1). Our data therefore supports the hypothesis of a coevolution in ligand specificity of elephant and horse PR on a molecular level.

**Figure 7:**
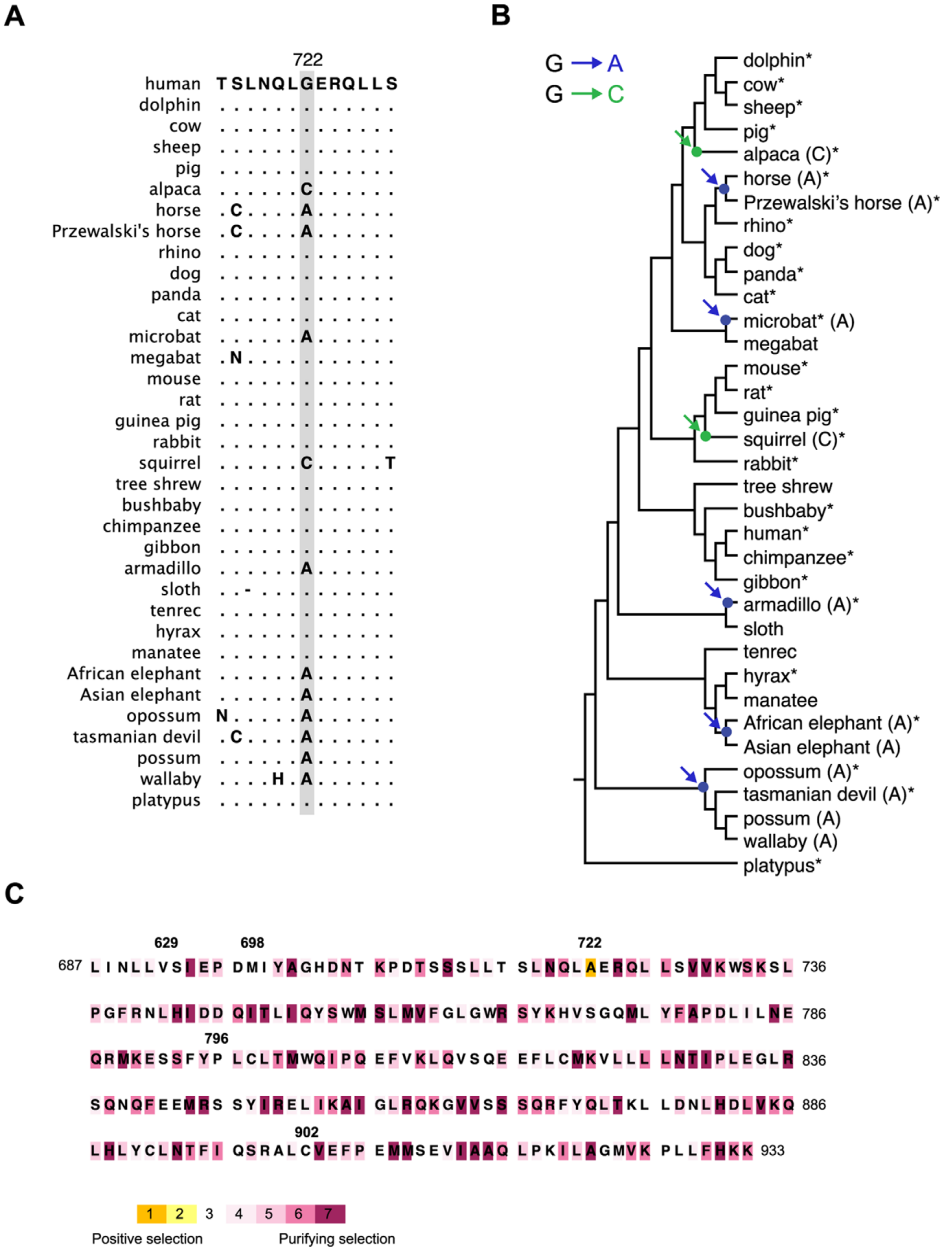
The G722A exchange evolved 5 times during mammalian evolution. (A) Sequence alignment of residue 722 and surrounding amino acids of the PR LBD. (B) Phylogenetic tree of mammalian evolution deduced from Murphy *et al.* (16) and Killian *et al.* (17). Blue arrows indicate the substitution of G722 to alanine, green arrows the substitution to cysteine. (C) PR LBD DNA sequences from mammals listed in (B) were aligned and a codon analysis for positive/purifying selection performed based on phylogenetic relationships depicted in (B). Residues of elephant PR are color-coded according to their selective pressure during mammalian evolution.

In order to address at which point in mammalian evolution the G722A exchange occurred, we analyzed the PR LBD sequences of the closest relatives of the African elephant and horse including Asian elephant, hyrax (*Procavia capensis*) and manatee (*Trichechus manatus*) as well as Przewalski’s horse (*Equus ferus przewalskii*) and rhino (*Ceratotherium simum simum*) respectively (Figure S1). As PR is exclusively expressed in reproductive target tissues, which are difficult to obtain for zoo species, we started from blood DNA and sequenced exons by taking use of degenerate primer pairs and deducing exon-intron boundaries by chromosome walking. Interestingly, apart from the Przewalski’s horse as the closest relative to *Equus caballus* carrying the G722A substitution, all other investigated species presented the Gly722 genotype (Figure 7A). We could therefore conclude that the G722A exchange in both elephants and horses appeared very late in evolution, before the separation of African and Asian elephant and before the upcoming of the Przewalski’s horse as the common ancestor of all extant horses (Figure 7B).

Having identified the G722A in two independent strains of mammalian evolution, we screened for the Ala722 phenotype in several other species of different classes. Surprisingly, the substitution was also present in all currently available PR sequences of marsupials including opossum, Tasmanian devil, possum and tammar wallaby, as well as in microbat and armadillo (Figure 7A). Altogether, the G722A exchange has occurred at least five times independently during mammalian evolution (Figure 7B). Additionally, we identified two independent G722C exchanges in the evolution of squirrel and alpaca indicating further evolutionary variation in this position (Figure 7B).

In order to define evolutionary selection forces underlying the G722A exchange and the other 4 elephant specific amino acid substitutions, we performed a phylogenetic codon analysis applying a combined empirical and mechanistic codon model [24], [25]. Interestingly, among all residues of PR LBD, residue 722 was the only one that revealed to be under positive selection (ω = 1.4), while the other 4 elephant specific exchanges had ω-values between 0.26 and 0.75 indicating neutral behavior during mammalian evolution (Figure 7C, Table S1). Comparing the results to an evolutionary null-model proofed the positive selection to be significant. We could therefore conclude that the substitution of Gly722 to alanine or cysteine evolved under positive selection and that this residue has a unique evolutionary standing among all other residues in the PR LBD.

## Discussion

In this study we analyzed the molecular basis of the different PR ligand-specificity of elephants compared to other mammalian species. We identified the G722A exchange to be responsible and sufficient to alter receptor specificity by specifically increasing affinity for DHP. Including the elephant, we found this substitution in the PR LBDs of mammalian species from five independent orders. Notably, the substitution was present in all currently available PR sequences of marsupials including opossum, Tasmanian devil, possum and tammar wallaby. In the latter, the G722A substitution has been described to play a possible in role in the resistance towards binding of the synthetic anti-gestagen RU486 [29]. This was deduced from a study with hamster and chicken PR, both bearing a G722C substitution, which specifically abolished the binding of RU486, while not affecting progesterone affinity [30]. Also elephants are known to be resistant towards the binding of RU486 indicating a similar role of resistance [31]. The presence of alanine at site 722 instead of glycine most likely prevents the formation of the RU486 binding pocket by a simple sterical clash of the alanine methyl side chain and the aromatic substituent at C11 of RU486 [29]. By showing that the G722A exchange also affects binding of an endogenous ligand, we now could firstly present a physiological role of substitutions at site 722 of PR.

Both progesterone and DHP contain identical side-chains and only differ in the presence or absence of a double bond at C5. The loss of this double bond in DHP leads to a conformational change in the A-ring conformation of the ligand. In the human PR the DHP A-ring thereby adopts a different position in the binding pocket affecting A-ring specific interactions. In the elephant PR, the different positioning of the DHP A-ring is blocked by the presence of the Ala722 methyl group, which would clash with the C1 of DHP and thereby pushes the DHP A-ring into a similar position than progesterone, explaining a similar binding affinity for both ligands.

Apart from the change in specificity, we observed that the elephant PR has a 2.3-fold higher binding affinity towards progesterone and DHP compared to the human hPR 722A mutant. The higher affinity of the elephant compared to human PR was partly mediated by the S796P exchange. However we found that introducing the S796P substitution in the G722A mutated human receptor did not lead to a further increase in affinity for neither DHP, nor progesterone. This could be explained by the finding that Leu796 neighboring the S796P exchange and binding the D-ring should be most sensitive to changes in position 722. Enhanced rigidity by either G722A or S796P leads to enhanced affinity, while the second substitution has no further effect. Hence, a possible evolutionary scenario would be that the S796P mutation occurred first in order to balance the restricted availability of progesterone, while the G722A exchange and with it the possibility to efficiently use DHP appeared in a later step during evolution. This theory is further strengthened by the fact, that also megabats acquired the S796P exchange independently of Ala722.

Both G722A and S796P substitutions also seem to be responsible for the different affinity profile of MGA, which is more bulky than progesterone. While the longer side chains of MGA result in a higher affinity to the human receptor, in the elephant PR they cause sterical clashes and thus drastically reduce affinity.

Steroid hormone receptors evolved under the principle of molecular exploitation [32]. The PR developed as a result of two rounds of gene duplication events, which starting from an ancestral estrogen receptor generated a functional progesterone and a corticosteroid receptor. As progesterone is an intermediate in the synthesis of estradiol, the duplicated receptor achieved specificity for a preexisting compound, known as “ligand first” model [32]. A third duplication event separated MR and GR from the ancestral corticosteroid receptor. In this case the ancestral receptor already had affinity for both mineralocorticoids and glucocorticoids, which was used by cortisol to build up a new receptor-hormone system, known as “receptor first” model [11].

For the evolution of ligand specificity of the elephant PR both “ligand first” and “receptor first” scenarios would be possible. However, having found the G722A exchange in several other mammalian species controlling pregnancy by progesterone including wallaby, armadillo and bat [33]–[35], the “receptor first” model would be the more likely. In this case, the 1.3-fold increase in progesterone affinity that we observed introducing G722A in the human PR would have been sufficient for positive selection of the mutation, followed by an opportunistic usage of the new ligand spectrum by horses and elephants, which resulted in a complete switch in hormone usage in the latter.

Interestingly, while the ligand specificity of horse and elephant evolved in parallel, the source of DHP synthesis differs for both species. In both African and Asian elephants DHP is directly synthesized in the corpora lutea of the ovaries by an unknown mechanism [5]. In horses, DHP is generated by 5-alpha reduction of progesterone in the placenta [27], [28]. The two different ways of taking advantage of the altered receptor specificity additionally supports the “receptor first” theory. Whether 5-alpha-reduced progestins play a role also in other mammalians carrying the Ala722 phenotype remains to be investigated.

## Supporting Information

Figure S1 **Comparison of human, horse and elephant PR LBD with sequenced PR LBD from related mammalian species.**
(PDF)

Table S1 **Output of the Selecton server analysis.**
(PDF)
