# Supplementary material for: A Single Glycine-Alanine Exchange Directs Ligand Specificity of the Elephant Progestin Receptor
Source: PLoS One. 2012 Nov 27;7(11):e50350. doi: 10.1371/journal.pone.0050350 (PMC3507690; doi:10.1371/journal.pone.0050350)
Supplement: Figure S1 — Comparison of human, horse and elephant PR LBD with sequenced PR LBD from related mammalian species. (PDF) [file pone.0050350.s001.pdf]

Supplemental Figure S1

```
                   687                                                736
human              LINLLMSIEP DVIYAGHDNT KPDTSSSLLT SLNQLGERQL LSVVKWSKSL
African elephant   .....V.... .M........ .......... .....A.... ..........
Asian elephant     .....V.... .M........ .......... .....A.... ..--------
manatee            .......... .M....Y... ..E....... .......... ..........
hyrax              .......... .M........ .......... .......... ...-------
rhino              .......... .......... .......... .......... ..........
horse              .......... .M........ .......... C....A.... ..........
Przwalski's horse  .......... .......... .......... C....A.... ..........

                                                                      786
human              PGFRNLHIDD QITLIQYSWM SLMVFGLGWR SYKHVSGQML YFAPDLILNE
African elephant   .......... .......... .......... .......... ..........
Asian elephant     ---------- ---------- ---------- ---------- ----------
manatee            .......... .......... .......... .......... ..........
hyrax              -......... .......... .......... .......... ..........
rhino              .......... .......... .......... .......... ..........
horse              ....K..... .......... .......... .......... ......V...
Przwalski's horse  .......... .......... .......... .......... ..........

                                                                      836
human              QRMKESSFYS LCLTMWQIPQ EFVKLQVSQE EFLCMKVLLL LNTIPLEGLR
African elephant   .........P .......... .......... .......... ..........
Asian elephant     .........P ........-- ---------- --........ ...-------
manatee            .......... .......... .......... .......... ..........
hyrax              .......... .......... .......... .......... ..........
rhino              .......... .......... .......... .......... ..........
horse              .......L.. .......... .......... .......... ..........
Przwalski's horse  .......L.. .......... .......... .......... ..........

                                                                      886
human              SQTQFEEMRS SYIRELIKAI GLRQKGVVSS SQRFYQLTKL LDNLHDLVKQ
African elephant   ..N....... .......... .......... .......... ..........
Asian elephant     --------.. .......... .......... .......--- ----------
manatee            ..N....... .......... .......... .......... ..........
hyrax              ..N....... .......... .......... .......... ..........
rhino              ..N....... .......... .......... .......... ..........
horse              ..N....... N......... .......... .........F ..T.......
Przwalski's horse  ..N....... N......... .......... .........F ..T.......

                                                                  933
human              LHLYCLNTFI QSRALSVEFP EMMSEVIAAQ LPKILAGMVK PLLFHKK
African elephant   .......... .....C.... .......... .......... .......
Asian elephant     ---------- ---------- ---------- ---------- -------
manatee            .......... .......... .......... .......... .......
hyrax              .......... .....C.... .......... .......... .......
rhino              .......... .......... .......... .......... .......
horse              .......... .......... ...T...... .......... .......
Przwalski's horse  .......... .......... ...T...... .......... .V.....
```

Supplemental Figure S1: Comparison of human, horse and elephant PR LBD with sequenced PR LBD from related mammalian species. PR LBD sequences of Asian elephant (*Elephas maximus*), manatee (*Trichechus manatus*), hyrax (*Procavia capensis*), Przewalski's horse (*Equus ferus przewalskii*) and rhino (*Ceratotherium simum simum*) were sequenced from genomic DNA and aligned to human, horse and elephant (*Loxodonta africana*) PR LBD. Shaded residues indicate elephant specific amino acid exchanges. Dots represent identical amino acids.
