# Supplementary material for: A Single Glycine-Alanine Exchange Directs Ligand Specificity of the Elephant Progestin Receptor
Source: PLoS One. 2012 Nov 27;7(11):e50350. doi: 10.1371/journal.pone.0050350 (PMC3507690; doi:10.1371/journal.pone.0050350)
Supplement: Table S1 — Output of the Selecton server analysis. (PDF) [file pone.0050350.s002.pdf]

**Supplemental Table S1: Output of the Selecton server analysis.** Bayesian Ka/Ks ratios derived from the mechanistic empirical combination model underlying the codon analysis of positive / purifying selection presented in Figure 7C. Ratios above 1 indicate positive selection of the codon.

| Pos. | Amino acid | Ka/Ks | [Confidence Interval] (* if lower bound > 1) |
|---|---|---|---|
| 687 | L | 0.024 | [3.3e-07,0.3] |
| 688 | I | 0.26 | [0.046,0.3] |
| 689 | N | 0.16 | [0.0041,0.3] |
| 690 | L | 0.024 | [3.3e-07,0.3] |
| 691 | L | 0.024 | [3.3e-07,0.3] |
| 692 | V | 0.3 | [0.3,0.3] |
| 693 | S | 0.29 | [0.046,0.3] |
| 694 | I | 0.011 | [3.3e-07,0.046] |
| 695 | E | 0.023 | [3.3e-07,0.3] |
| 696 | P | 0.023 | [3.3e-07,0.3] |
| 697 | D | 0.17 | [0.0041,0.3] |
| 698 | M | 0.65 | [0.3,1] |
| 699 | I | 0.76 | [0.3,1] |
| 700 | Y | 0.022 | [3.3e-07,0.3] |
| 701 | A | 0.013 | [3.3e-07,0.046] |
| 702 | G | 0.036 | [3.3e-07,0.3] |
| 703 | H | 0.25 | [0.046,0.3] |
| 704 | D | 0.024 | [3.3e-07,0.3] |
| 705 | N | 0.018 | [3.3e-07,0.3] |
| 706 | T | 0.3 | [0.046,0.3] |
| 707 | K | 0.018 | [3.3e-07,0.3] |
| 708 | P | 0.025 | [3.3e-07,0.3] |
| 709 | D | 0.31 | [0.046,1] |
| 710 | T | 0.015 | [3.3e-07,0.046] |
| 711 | S | 0.18 | [0.0041,0.3] |
| 712 | S | 0.01 | [3.3e-07,0.046] |
| 713 | S | 0.13 | [0.0041,0.3] |
| 714 | L | 0.021 | [3.3e-07,0.3] |
| 715 | L | 0.023 | [3.3e-07,0.3] |
| 716 | T | 0.14 | [0.0041,0.3] |
| 717 | S | 0.2 | [0.046,0.3] |
| 718 | L | 0.021 | [3.3e-07,0.3] |
| 719 | N | 0.013 | [3.3e-07,0.046] |
| 720 | Q | 0.14 | [0.0041,0.3] |
| 721 | L | 0.023 | [3.3e-07,0.3] |
| 722 | A | 1.4 | [1.4,1.4]* |
| 723 | E | 0.023 | [3.3e-07,0.3] |
| 724 | R | 0.012 | [3.3e-07,0.046] |
| 725 | Q | 0.015 | [3.3e-07,0.046] |
| 726 | L | 0.019 | [3.3e-07,0.3] |
| 727 | L | 0.018 | [3.3e-07,0.3] |
| 728 | S | 0.13 | [0.0041,0.3] |
| 729 | V | 0.0093 | [3.3e-07,0.046] |
| 730 | V | 0.013 | [3.3e-07,0.046] |
| 731 | K | 0.018 | [3.3e-07,0.3] |
| 732 | W | 0.048 | [3.3e-07,0.3] |
| 733 | S | 0.015 | [3.3e-07,0.046] |
| 734 | K | 0.017 | [3.3e-07,0.3] |
| 735 | S | 0.35 | [0.3,1] |
| 736 | L | 0.024 | [3.3e-07,0.3] |
| 737 | P | 0.022 | [3.3e-07,0.3] |
| 738 | G | 0.026 | [3.3e-07,0.3] |
| 739 | F | 0.02 | [3.3e-07,0.3] |
| 740 | R | 0.022 | [3.3e-07,0.3] |
| 741 | N | 0.15 | [0.0041,0.3] |
| 742 | L | 0.022 | [3.3e-07,0.3] |
| 743 | H | 0.013 | [3.3e-07,0.046] |
| 744 | I | 0.011 | [3.3e-07,0.046] |
| 745 | D | 0.019 | [3.3e-07,0.3] |
| 746 | D | 0.022 | [3.3e-07,0.3] |
| 747 | Q | 0.016 | [3.3e-07,0.046] |
| 748 | I | 0.0089 | [3.3e-07,0.046] |

| | | | |
|---|---|---|---|
| 749 | T | 0.011 | [3.3e-07,0.046] |
| 750 | L | 0.025 | [3.3e-07,0.3] |
| 751 | I | 0.014 | [3.3e-07,0.046] |
| 752 | Q | 0.016 | [3.3e-07,0.046] |
| 753 | Y | 0.023 | [3.3e-07,0.3] |
| 754 | S | 0.015 | [3.3e-07,0.046] |
| 755 | W | 0.048 | [3.3e-07,0.3] |
| 756 | M | 0.01 | [3.3e-07,0.046] |
| 757 | S | 0.012 | [3.3e-07,0.046] |
| 758 | L | 0.023 | [3.3e-07,0.3] |
| 759 | M | 0.01 | [3.3e-07,0.046] |
| 760 | V | 0.014 | [3.3e-07,0.046] |
| 761 | F | 0.019 | [3.3e-07,0.3] |
| 762 | G | 0.53 | [0.046,1] |
| 763 | L | 0.024 | [3.3e-07,0.3] |
| 764 | G | 0.035 | [3.3e-07,0.3] |
| 765 | W | 0.048 | [3.3e-07,0.3] |
| 766 | R | 0.012 | [3.3e-07,0.046] |
| 767 | S | 0.018 | [3.3e-07,0.3] |
| 768 | Y | 0.024 | [3.3e-07,0.3] |
| 769 | K | 0.017 | [3.3e-07,0.3] |
| 770 | H | 0.12 | [0.0041,0.3] |
| 771 | V | 0.13 | [0.0041,0.3] |
| 772 | S | 0.079 | [0.0041,0.3] |
| 773 | G | 0.26 | [0.0041,1] |
| 774 | Q | 0.26 | [0.046,0.3] |
| 775 | M | 0.011 | [3.3e-07,0.046] |
| 776 | L | 0.024 | [3.3e-07,0.3] |
| 777 | Y | 0.022 | [3.3e-07,0.3] |
| 778 | F | 0.019 | [3.3e-07,0.3] |
| 779 | A | 0.013 | [3.3e-07,0.046] |
| 780 | P | 0.023 | [3.3e-07,0.3] |
| 781 | D | 0.019 | [3.3e-07,0.3] |
| 782 | L | 0.024 | [3.3e-07,0.3] |
| 783 | I | 0.078 | [0.0041,0.3] |
| 784 | L | 0.33 | [0.046,1] |
| 785 | N | 0.013 | [3.3e-07,0.046] |
| 786 | E | 0.024 | [3.3e-07,0.3] |
| 787 | Q | 0.017 | [3.3e-07,0.046] |
| 788 | R | 0.021 | [3.3e-07,0.3] |
| 789 | M | 0.011 | [3.3e-07,0.046] |
| 790 | K | 0.019 | [3.3e-07,0.3] |
| 791 | E | 0.024 | [3.3e-07,0.3] |
| 792 | S | 0.41 | [0.3,1] |
| 793 | S | 0.016 | [3.3e-07,0.046] |
| 794 | F | 0.37 | [0.046,1] |
| 795 | Y | 0.024 | [3.3e-07,0.3] |
| 796 | P | 0.26 | [0.046,0.3] |
| 797 | L | 0.024 | [3.3e-07,0.3] |
| 798 | C | 0.038 | [3.3e-07,0.3] |
| 799 | L | 0.02 | [3.3e-07,0.3] |
| 800 | T | 0.017 | [3.3e-07,0.046] |
| 801 | M | 0.011 | [3.3e-07,0.046] |
| 802 | W | 0.052 | [3.3e-07,0.3] |
| 803 | Q | 0.017 | [3.3e-07,0.046] |
| 804 | I | 0.016 | [3.3e-07,0.046] |
| 805 | P | 0.024 | [3.3e-07,0.3] |
| 806 | Q | 0.017 | [3.3e-07,0.3] |
| 807 | E | 0.025 | [3.3e-07,0.3] |
| 808 | F | 0.021 | [3.3e-07,0.3] |
| 809 | V | 0.16 | [0.0041,0.3] |
| 810 | K | 0.02 | [3.3e-07,0.3] |
| 811 | L | 0.02 | [3.3e-07,0.3] |
| 812 | Q | 0.016 | [3.3e-07,0.046] |
| 813 | V | 0.28 | [0.046,0.3] |
| 814 | S | 0.26 | [0.046,0.3] |
| 815 | Q | 0.56 | [0.3,1] |
| 816 | E | 0.024 | [3.3e-07,0.3] |
| 817 | E | 0.025 | [3.3e-07,0.3] |
| 818 | F | 0.026 | [3.3e-07,0.3] |
| 819 | L | 0.028 | [3.3e-07,0.3] |

| | | | |
|---|---|---|---|
| 820 | C | 0.03 | [3.3e-07,0.3] |
| 821 | M | 0.011 | [3.3e-07,0.046] |
| 822 | K | 0.019 | [3.3e-07,0.3] |
| 823 | V | 0.21 | [0.046,0.3] |
| 824 | L | 0.023 | [3.3e-07,0.3] |
| 825 | L | 0.025 | [3.3e-07,0.3] |
| 826 | L | 0.02 | [3.3e-07,0.3] |
| 827 | L | 0.02 | [3.3e-07,0.3] |
| 828 | N | 0.015 | [3.3e-07,0.046] |
| 829 | T | 0.016 | [3.3e-07,0.046] |
| 830 | I | 0.012 | [3.3e-07,0.046] |
| 831 | P | 0.024 | [3.3e-07,0.3] |
| 832 | L | 0.023 | [3.3e-07,0.3] |
| 833 | E | 0.024 | [3.3e-07,0.3] |
| 834 | G | 0.037 | [3.3e-07,0.3] |
| 835 | L | 0.025 | [3.3e-07,0.3] |
| 836 | R | 0.013 | [3.3e-07,0.046] |
| 837 | S | 0.088 | [0.0041,0.3] |
| 838 | Q | 0.016 | [3.3e-07,0.046] |
| 839 | N | 1 | [0.3,1] |
| 840 | Q | 0.017 | [3.3e-07,0.046] |
| 841 | F | 0.02 | [3.3e-07,0.3] |
| 842 | E | 0.9 | [0.3,1] |
| 843 | E | 0.2 | [0.0041,0.3] |
| 844 | M | 0.011 | [3.3e-07,0.046] |
| 845 | R | 0.012 | [3.3e-07,0.046] |
| 846 | S | 0.38 | [0.3,1] |
| 847 | S | 0.29 | [0.046,0.3] |
| 848 | Y | 0.03 | [3.3e-07,0.3] |
| 849 | I | 0.011 | [3.3e-07,0.046] |
| 850 | R | 0.013 | [3.3e-07,0.046] |
| 851 | E | 0.025 | [3.3e-07,0.3] |
| 852 | L | 0.027 | [3.3e-07,0.3] |
| 853 | I | 0.016 | [3.3e-07,0.046] |
| 854 | K | 0.019 | [3.3e-07,0.3] |
| 855 | A | 0.014 | [3.3e-07,0.046] |
| 856 | I | 0.011 | [3.3e-07,0.046] |
| 857 | G | 0.026 | [3.3e-07,0.3] |
| 858 | L | 0.022 | [3.3e-07,0.3] |
| 859 | R | 0.013 | [3.3e-07,0.046] |
| 860 | Q | 0.016 | [3.3e-07,0.046] |
| 861 | K | 0.018 | [3.3e-07,0.3] |
| 862 | G | 0.29 | [0.0041,1] |
| 863 | V | 0.01 | [3.3e-07,0.046] |
| 864 | V | 0.013 | [3.3e-07,0.046] |
| 865 | S | 0.46 | [0.3,1] |
| 866 | S | 0.013 | [3.3e-07,0.046] |
| 867 | S | 0.015 | [3.3e-07,0.046] |
| 868 | Q | 0.017 | [3.3e-07,0.046] |
| 869 | R | 0.02 | [3.3e-07,0.3] |
| 870 | F | 0.026 | [3.3e-07,0.3] |
| 871 | Y | 0.024 | [3.3e-07,0.3] |
| 872 | Q | 0.015 | [3.3e-07,0.046] |
| 873 | L | 0.019 | [3.3e-07,0.3] |
| 874 | T | 0.015 | [3.3e-07,0.046] |
| 875 | K | 0.17 | [0.0041,0.3] |
| 876 | L | 0.6 | [0.3,1] |
| 877 | L | 0.3 | [0.046,1] |
| 878 | D | 0.021 | [3.3e-07,0.3] |
| 879 | N | 0.48 | [0.3,1] |
| 880 | L | 0.022 | [3.3e-07,0.3] |
| 881 | H | 0.013 | [3.3e-07,0.046] |
| 882 | D | 0.02 | [3.3e-07,0.3] |
| 883 | L | 0.02 | [3.3e-07,0.3] |
| 884 | V | 0.014 | [3.3e-07,0.046] |
| 885 | K | 0.018 | [3.3e-07,0.3] |
| 886 | Q | 0.016 | [3.3e-07,0.046] |
| 887 | L | 0.02 | [3.3e-07,0.3] |
| 888 | H | 0.013 | [3.3e-07,0.046] |
| 889 | L | 0.025 | [3.3e-07,0.3] |
| 890 | Y | 0.031 | [3.3e-07,0.3] |

| | | | |
|---|---|---|---|
| 891 | C | 0.037 | [3.3e-07,0.3] |
| 892 | L | 0.023 | [3.3e-07,0.3] |
| 893 | N | 0.015 | [3.3e-07,0.046] |
| 894 | T | 0.015 | [3.3e-07,0.046] |
| 895 | F | 0.021 | [3.3e-07,0.3] |
| 896 | I | 0.016 | [3.3e-07,0.046] |
| 897 | Q | 0.17 | [0.0041,0.3] |
| 898 | S | 0.022 | [3.3e-07,0.3] |
| 899 | R | 0.026 | [3.3e-07,0.3] |
| 900 | A | 0.25 | [0.046,0.3] |
| 901 | L | 0.025 | [3.3e-07,0.3] |
| 902 | C | 0.75 | [0.3,1] |
| 903 | V | 0.011 | [3.3e-07,0.046] |
| 904 | E | 0.024 | [3.3e-07,0.3] |
| 905 | F | 0.021 | [3.3e-07,0.3] |
| 906 | P | 0.023 | [3.3e-07,0.3] |
| 907 | E | 0.024 | [3.3e-07,0.3] |
| 908 | M | 0.011 | [3.3e-07,0.046] |
| 909 | M | 0.011 | [3.3e-07,0.046] |
| 910 | S | 0.14 | [0.0041,0.3] |
| 911 | E | 0.19 | [0.0041,0.3] |
| 912 | V | 0.086 | [0.0041,0.3] |
| 913 | I | 0.011 | [3.3e-07,0.046] |
| 914 | A | 0.014 | [3.3e-07,0.046] |
| 915 | A | 0.015 | [3.3e-07,0.046] |
| 916 | Q | 0.016 | [3.3e-07,0.046] |
| 917 | L | 0.023 | [3.3e-07,0.3] |
| 918 | P | 0.033 | [3.3e-07,0.3] |
| 919 | K | 0.17 | [0.0041,0.3] |
| 920 | I | 0.016 | [3.3e-07,0.046] |
| 921 | L | 0.023 | [3.3e-07,0.3] |
| 922 | A | 0.014 | [3.3e-07,0.046] |
| 923 | G | 0.3 | [0.0041,1] |
| 924 | M | 0.098 | [0.0041,0.3] |
| 925 | V | 0.018 | [3.3e-07,0.3] |
| 926 | K | 0.019 | [3.3e-07,0.3] |
| 927 | P | 0.32 | [0.046,1] |
| 928 | L | 0.18 | [0.0041,0.3] |
| 929 | L | 0.26 | [0.0041,1] |
| 930 | F | 0.02 | [3.3e-07,0.3] |
| 931 | H | 0.014 | [3.3e-07,0.046] |
| 932 | K | 0.16 | [0.0041,0.3] |
| 933 | K | 0.019 | [3.3e-07,0.3] |
